# Modeling protein quaternary structure of homo- and hetero-oligomers beyond binary interactions by homology

**DOI:** 10.1038/s41598-017-09654-8

**Published:** 2017-09-05

**Authors:** Martino Bertoni, Florian Kiefer, Marco Biasini, Lorenza Bordoli, Torsten Schwede

**Affiliations:** 1 SIB Swiss Institute of Bioinformatics, Basel, Switzerland; 2 Biozentrum, University of Basel, Klingelbergstrasse 50/70, 4056 Basel, Switzerland

## Abstract

Cellular processes often depend on interactions between proteins and the formation of macromolecular complexes. The impairment of such interactions can lead to deregulation of pathways resulting in disease states, and it is hence crucial to gain insights into the nature of macromolecular assemblies. Detailed structural knowledge about complexes and protein-protein interactions is growing, but experimentally determined three-dimensional multimeric assemblies are outnumbered by complexes supported by non-structural experimental evidence. Here, we aim to fill this gap by modeling multimeric structures by homology, only using amino acid sequences to infer the stoichiometry and the overall structure of the assembly. We ask which properties of proteins within a family can assist in the prediction of correct quaternary structure. Specifically, we introduce a description of protein-protein interface conservation as a function of evolutionary distance to reduce the noise in deep multiple sequence alignments. We also define a distance measure to structurally compare homologous multimeric protein complexes. This allows us to hierarchically cluster protein structures and quantify the diversity of alternative biological assemblies known today. We find that a combination of conservation scores, structural clustering, and classical interface descriptors, can improve the selection of homologous protein templates leading to reliable models of protein complexes.

## Introduction

Macromolecular complexes are of central interest in structural biology^1–3^. Direct physical protein-protein interactions (PPIs), as well as indirect ones, are essential for performing and regulating cellular activities such as signal transduction, cell-cycle, morphological differentiation, cell motility, transcription and translation. A precise description of proteins’ interactions and quaternary structure (QS) is fundamental to gain a detailed molecular understanding on how these interactions are mediated and regulated. While experimental information on interacting partners obtained with high-throughput methods^4–6^ such as two-hybrid screening (Y2H) or affinity purification of complexes grows with an exponential trend^7–10^, the number of experimentally determined three-dimensional complexes and oligomeric structures is lagging behind. Shedding light on the atomic details of such interactions is challenging since the expression of protein complexes is often tightly regulated and obtaining sufficient concentrations of intact complexes for structure determination is often not trivial.

Aiming to fill this gap, several computational techniques to model protein interactions have been developed, which differ in type and amount of structural information required as starting point. One of the first approaches used to model interactions *de novo*, when structures of the individual components are available, was macromolecular docking. The relative orientation of two proteins is sampled and scored by exploiting e.g. the components’ shape^11^ or physicochemical complementarity^12^. Recently, amino acid co-evolution analysis (see ref. 13 for a review) has been successfully applied to identify proximal residues in interfaces^14^ thus increasing the accuracy of the results. Docking approaches are generally more accurate when no significant conformational changes are required for interface formation, as documented by the regular CAPRI experiment (Critical Assessment of Prediction of Interactions)^15^. When some experimental details of the interaction are available (e.g. EM density maps, crosslinking, SAXS or NMR data, co-evolution analysis, etc.), different “hybrid-modeling” tools can be used (e.g. the Integrative Modeling Platform (IMP)^16^, the Rosetta Suite^17^, or HADDOCK^18^) to apply experimental constrains when modeling sizable assemblies.

The number of ways proteins interact in nature is probably limited^19, 20^, and it has been observed that similar binding modes can be identified for almost all known protein-protein interactions^21^. Furthermore, Honig’s group noted that the location of the interface in structural homologs is often conserved^22^. These observations paved the way for homology modeling (aka comparative or template-based modeling) of protein complexes, where uncharacterized interactions are modeled using experimental structures of homologous interacting protomers (interologs) as templates. Approaches based on homology are scalable to full genomes and successfully reduced the gap between known interactions and those that are structurally characterized for several practical applications^21, 23–25^.

While some *in silico* docking techniques exploit information about the stoichiometry or the symmetry of the complex^26–29^ to predict multimeric assemblies, the majority of docking and homology based approaches are focused on dimeric interactions, bypassing higher-order quaternary structures. The importance of prediction of complex assemblies has been highlighted by the introduction of quaternary structure prediction assessment in the recent CASP XII (Critical Assessment of protein Structure Prediction)^30, 31^ and the CAMEO (Continuous Automated Model Evaluation)^32^ experiments. In this study, we propose an approach to identify the stoichiometry and overall structure of protein complexes using amino acid sequences as starting point. We focus on efficiently using the information on quaternary structures available in the PDB repository and encoded in multiple sequence alignments for extending the scope and automating homology modeling to appropriately address protein assemblies.

Overall, throughout a given protein family quaternary structure is less conserved than tertiary structure, i.e. while the fold of a polypeptide chain remains structurally similar the number of subunits forming the biologically relevant quaternary structure can vary significantly^33, 34^. However, if a specific interaction between two protein chains plays a structural or functional role, it is reasonable to expect that residues at the corresponding interface are less free to vary hence increasing evolutionary conservation in these regions^35, 36^. Here, we introduce a refined analysis of interface conservation which captures how interface conservation varies as a function of evolutionary distance within a protein family. We employ this analysis (which we refer to as Protein-Protein Interaction (PPI) fingerprints) for two critical tasks: first, the discrimination of crystal artifacts from biological contacts, which is a crucial step in determining the correct quaternary state of crystal structures to be used as templates in homology modeling; and second, the evaluation of interface quality in models to assess the confidence in the predicted quaternary structure.

In parallel to these evolutionary considerations we also analyze the geometry of oligomers. Even at high sequence identity, proteins are often represented in multiple different conformations and quaternary structures in the PDB. Hence, selecting correct templates for homology modeling is essential. We define a distance measure (QS-score) that quantifies the similarity between interfaces as a function of shared interfacial contacts. QS-score thereby discriminates between alternative quaternary structures and binding modes. We use this distance measure to evaluate the diversity of quaternary conformations represented in experimental structures and for measuring the accuracy of models.

Using a supervised machine learning approach, Support Vector Machines (SVM), we combine interface conservation, structural clustering and other template features to rank and automatically select templates that maximize the predicted interface quality for a specific protein of interest. Based on this approach we were able to assign the correct quaternary structure for the majority of proteins of our data set. Finally, the application of our approach is illustrated by the prediction of fructose bisphosphate aldolase (FBA) from *Haloferax volcanii*, which exemplifies the modeling challenges faced when homologs in closely related organisms assume a variety of oligomeric conformations.

## Results and Discussion

### Interface conservation: PPI fingerprints

Proteins acquire oligomeric organization for a variety of functional and biophysical advantages: modular elements are less prone to coding errors, oligomeric regulation add an additional level of control, large structures are more stable and can perform their function cooperatively^37^. These and other processes are influencing the evolution of proteins’ interface formation^34, 38^. During evolution, different mechanisms can modify a proteins oligomeric state: direct mutations occurring at the subunit interface or indirect mutations allosterically inducing a change in binding modes^39^. Several groups have analyzed the impact of evolutionary pressure on protein-protein interfaces^36, 40, 41^. These analyses rely on an estimation of conservation that is typically derived from a multiple sequence alignment (MSA) of homologous proteins. Residues participating in interfaces are subject to different evolutionary constraints than residues at the protein surface interacting with the solvent, which creates a confounding factor when proteins organized in different quaternary structures are included in the same alignment.

**Figure 1:**
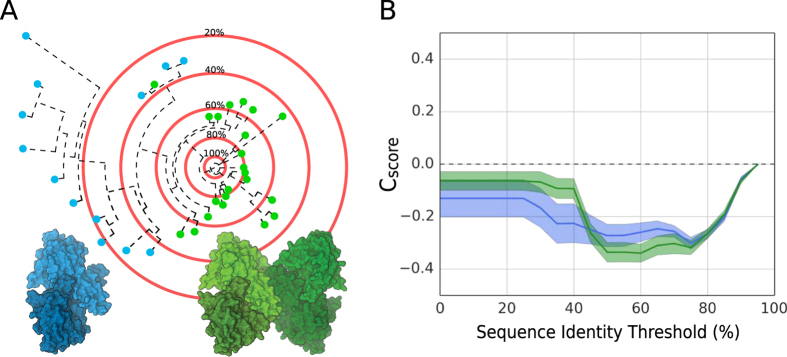
PPI Fingerprint concept. (**A**) The idealized sequence space of fructose bisphosphate aldolase represented as a phylogenetic tree rooted on a specific sequence. In this family of proteins, we observe either dimeric (blue) or tetrameric quaternary structures (green). The red concentric circles represent the sequence identity thresholds used to calculate the interface conservation score (C_score_). (**B**) The PPI fingerprint curves of several homologs with dimeric (blue) or tetrameric (green) quaternary structures (standard error is used for the error area). The MSA is obtained running HHblits^42^ against the non-redundant (20% sequence identity) NCBI database with a threshold of 70% as minimum coverage. Considering the complete MSA (below 20% sequence identity threshold) the support for a conserved interface is stronger for dimers, while with more stringent threshold (50–60%) the tetrameric option has a stronger conservation signal.

We expose this confounding factor in our conservation analysis by expressing the ratio between interface and surface residue entropy as a function of evolutionary distance as exemplified in Fig. 1 (see “Conservation Score” in Materials and Methods). For example, the fructose bisphosphate aldolase family consists of a mixture of dimers and tetramers (blue and green dots in Fig. 1A). The resulting conservation score curves (Fig. 1B) have values below zero indicating a higher mutation rate of surface residues compared to those at the interface, confirming the interface conservation of the protein family.

We refer to these family specific curves as PPI fingerprints as they capture the impact of evolutionary pressure on protein-protein interaction sites. The curves follow a characteristic pattern: when only very similar sequences are considered (80–90% sequence identity thresholds) the ratio is close to zero since the low variability in the MSA provides little information on the interface conservation. As we lower the inclusion threshold, the indication for a conserved interface is stronger and eventually reaches a minimum (at around 60% sequence identity in our example). When including remote homologs, the ratio tends back to zero, indicating that the signal is weakened by poorly conserved residues in the interface due to inclusion of proteins with different arrangements. In the example shown in Fig. 1, when more remote homologs below 40% sequence identity are included, the dimers’ curve has a stronger conservation signal then the tetramers’ one, while including only close homologs (above 60% sequence identity) the picture changes and the stronger evolutionary support is attributed to the tetramers. That is, alternative oligomeric states will have different PPI fingerprints and thus provide additional criterion for quaternary structure prediction.

A simple validation for our approach is to check whether PPI fingerprints help to discriminate between crystal contacts and biologically relevant protein interactions. Crystal contacts are protein-protein interfaces derived from the tight packing of proteins in crystals and should not carry any conservation signal. On the contrary, we expect evolutionary pressure to act on biological interfaces to maintain the function of the complex.

**Figure 2:**
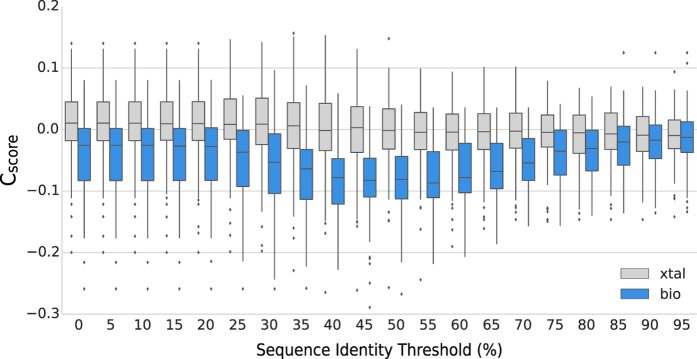
PPI fingerprints of the proteins in the Duarte *et al*. dataset. 83 biological interfaces (bio) are shown in blue, 82 crystal contacts (xtal) in grey. We see how the conservation score (y-axis), computed on MSAs generated with different sequence identity inclusion thresholds (x-axis), is helping to discriminate between crystal contacts and biological relevant interfaces. Using an inclusive MSA (0–25% sequence identity thresholds) the two non-normal distributions overlap to a large extent (Mann-Whitney p-values between 8.12 × 10^−7^ and 3.82 × 10^−8^), while in the threshold range between 35–55% they are clearly separable (Mann-Whitney p-values between 7.47 × 10^−11^ and 4.56 × 10^−13^).

We computed the PPI fingerprint curves on a recent manually curated dataset of interactions^43^. This dataset is composed of the two classes of protein contacts: crystal artifacts (82 interfaces), and biological contacts (83 interfaces). The dataset was created with stringent crystallographic quality criteria, including only experimentally confirmed quaternary structures, and focusing on small interfaces (up to 2000 Å^2^) where the discrimination is more difficult. Our results indicate that PPI fingerprints calculated from the crystal contacts group have a constant median around zero, while in the biologically relevant class we clearly observe a significant shift towards negative values (Fig. 2). We compared the conservation score distributions for crystal and biological interfaces using the Mann-Whitney test: the p-values for distributions between 35–55% inclusion thresholds are significantly lower than those obtained using the full MSA, in agreement with the finding by Duarte *et al*.^43^.

### Interface similarity: QS-score

In order to measure the structural similarity of protein-protein interfaces, several methods have been developed in recent years^15, 33, 44–51^ (summarized in Supplementary Table S1). Distance metrics developed in the context of protein-protein docking are mainly focusing on binary interactions. However, decomposing the comparison of assemblies into binary interactions can result in a factorial number of comparisons and missing interfaces (e.g. comparing a dimer to a tetramer) remain unaccounted.

For describing the diversity of quaternary structures represented in PDB we have developed QS-score as a distance measure, inspired by Q-score^44, 45^, which overcomes these limitations. QS-score considers the assembly interface as a whole and is suitable for comparing homo- or hetero-oligomers with identical or different stoichiometries, alternative relative orientations of chains, and distinct amino acid sequences (i.e. homologous complexes). To unequivocally identify the residues of all protein chains in complexes, QS-score first establishes a mapping between equivalent polypeptide chains of the compared structures (see “QS-score Algorithm” in Materials and Methods). QS-score expresses the fraction of shared interface contacts (residues on different chains with a Cβ-Cβ distance < 12 Å) between two assemblies. When the QS-score is close to 1 it indicates that the compared interfaces are very similar, so the complexes have equal stoichiometry and a majority of the interfacial contacts are identical. On the other end, a QS-score close to 0 indicates a radically diverse quaternary structure, so the assemblies may have different stoichiometries and/or may represent alternative binding conformations.

**Figure 3:**
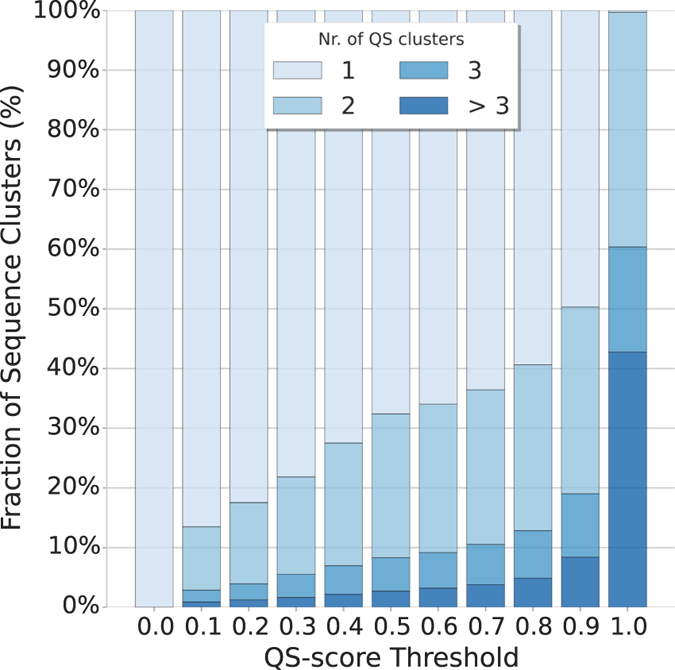
Heterogeneity of quaternary structures available in the Protein Data Bank (PDB). Assemblies from the PDB were clustered by sequence identity (90% sequence identity). All the assemblies within one sequence cluster were compared using QS-score. The resulting distance matrix was used to perform hierarchical clustering using different distance thresholds. With a distance threshold (x-axis) of 0 all assemblies are clustered together so that the fraction of sequence clusters (y-axis) having only one QS cluster is 100%. As the threshold is increased the structural heterogeneity of the sequence clusters is evident and the fraction of sequence clusters having multiple QS clusters (in shades of blue) increases.

We used QS-score to analyze the structural heterogeneity of all homo- and hetero-oligomeric assemblies deposited in the PDB. Sequences were clustered into groups sharing more than 90% sequence identity and for each sequence cluster we performed structural hierarchical clustering using different QS-score thresholds (see “PDB-wide QS clustering” in Material and Methods). Figure 3 shows the fraction of sequence clusters being homogeneous (with a single QS cluster) or heterogeneous (with two or more QS clusters). Even at this high level of sequence identity, the analysis shows that sequence neighbors do not always exhibit structurally identical interfaces. Using a QS-score threshold of 0.5, hence grouping structures having similar interfaces and identical stoichiometry, one third of the sequence clusters contain assemblies with interfaces different from each other.

This structural interface diversity between assemblies sharing high sequence identity represents a challenge for inferring the quaternary structure by homology considerations. All alternative QS options must be considered as potential templates in a protein structure homology modeling approach since a decision based on sequence similarity cannot distinguish between different oligomeric conformations. In order to choose the most suitable template for modeling, we analyzed several features of the target-template pairs as discussed in the following paragraphs.

### Homology modeling of oligomers

Here, we aim to extend the classical protein structure homology approach, which is typically applied to model single protein chains based on a target-template sequence alignment, to a generic quaternary structure modeling method by exploiting structural information available from homologous complexes. To identify suitable templates for the target protein(s), we apply the following criteria: each target sequence must have at least one homologous chain in the template; different target sequences cannot refer to overlapping fragments of the same chain in the template; the heteromeric template must be topologically connected, i.e. chains must physically interact to form a complex.

**Figure 4:**
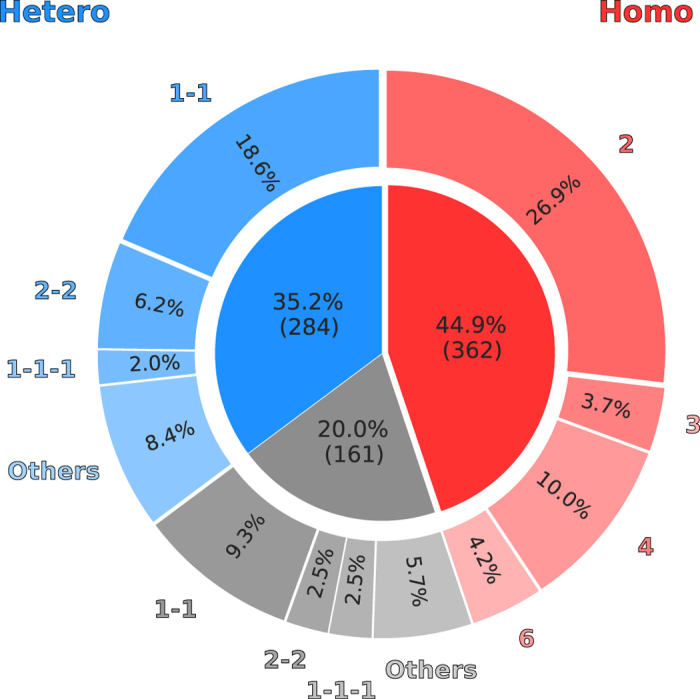
Stoichiometry of 807 target proteins in the TARGET dataset. Homo-oligomers are represented in shades of red, while hetero-oligomers in shades of blue. In shades of gray are the heteromeric targets for which no template could be identified. Each wedge of the pie chart is annotated with the fraction of the total dataset for the most common stoichiometries.

We compiled a dataset (TARGET) of 807 non-redundant proteins with experimentally validated quaternary structures (see “TARGET Dataset” in Materials and Methods). This balanced dataset is composed of 362 homo- and 445 hetero-oligomers of varying stoichiometries as reported in Fig. 4. For each of the TARGET dataset proteins we performed an extensive template search against the SWISS-MODEL template library^52^. To avoid bias introduced by close variants of the target proteins, we removed target-template pairs having a sequence identity higher than 95%. The largest fraction of complexes deposited in the PDB – which as of the time of this analysis contains about 120,000 entries – is composed of homo-oligomers, with more than 40,000 entries, whereas hetero-complexes are scarcer, in the order of 14,000 structures. It is hence not surprising that for all homomeric targets at least one homologous template could be identified, while for 36% (161) of the heteromeric targets no homologous complex was identified.

**Figure 5:**
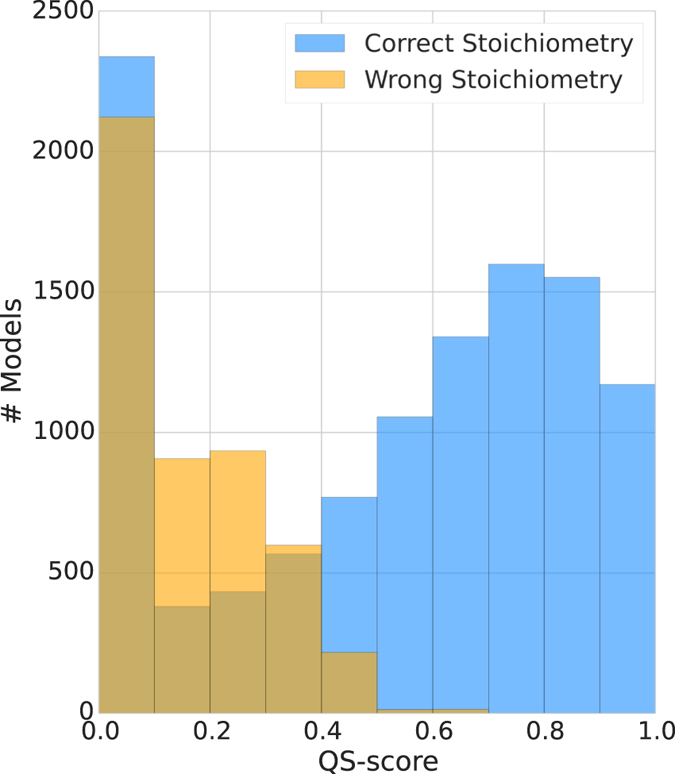
QS-score distribution for all generated models compared to the native structure. For both, model with a correct (blue) or incorrect (yellow) stoichiometry, a sizable fraction of models have an interface different from the native one as they are based on a template having a different, i.e. incorrect quaternary structure.

All potential templates were then used to generate models of the target protein and collected in our MODEL dataset (see “MODEL Dataset” in Materials and Methods). Since for each model, the experimental reference structure is known, we can directly compare and measure their QS-score to the native structure (i.e. the fraction of correctly modeled interface residues). The accuracy of the resulting models is reported in Fig. 5. Models with an incorrect stoichiometry have QS-scores consistently below 0.5 while correct stoichiometries distribute preferentially toward high QS-scores values peaking at around 0.7. The number of completely incorrect models with very low QS-score is high, emphasizing the importance of ranking the templates and favoring those leading to correctly modeled interfaces.

### Template ranking by quality prediction

Machine learning techniques have been frequently adopted in the context of quaternary structure prediction and preeminently applied to the problem of discriminating crystal vs. biological contacts^53–55^ and for the prediction of PPI interfaces^56^. In this study, we employ a supervised learning approach using Support Vector Machines (SVM) to predict the expected model-target QS-score given a set of template features. SVMs are scalable to large datasets and they can capture non-linear relationships using kernel functions.

The complete dataset that will be used for machine learning is composed of more than 20,000 models for a total of 645 different complexes. Our aim is to identify which features of the obtained target-template alignment would aid in the selection of templates leading to a correct quaternary structure model. For this purpose we measure four kinds of properties: (1) sequence properties, (2) MSA properties, (3) QS consensus properties and (4) interface composition properties. Sequence properties include sequence identity and similarity (BLOSUM62 based) of the target-template alignment, and an agreement measure of secondary structure and solvent accessibility prediction. These features are computed considering the different structural regions of the template: (i) the entire structure, (ii) the template’s interface residues, (iii) the core residues, and (iv) the surface residues. The MSA properties are derived from the target’s family multiple sequence alignment. These include average profile entropy and the template e-value obtained from the HHblits^42^ run as well as the PPI fingerprint (see above). For the latter, we rely on the template interface fraction that is mapped on the target sequence for which we compute the PPI fingerprint curve. We represent the resulting PPI fingerprint curve by the minimum of the curve, its area, the absolute maximum, and the conservation score obtained considering the full MSA. To derive QS consensus properties, we first cluster templates hierarchically by (i) oligomeric state (i.e. being monomers, homo- or hetero-oligomers), by (ii) stoichiometry and by (iii) geometry using the QS-score measure (see “Clustering Homologous Assemblies” in Materials and Methods). The QS consensus properties are then calculated as a template’s cluster size relative to the total number of homologs considering the different levels (i-iii) of clustering. Composition features are defined as in ref. 57 by comparing the relative hydrophobic and hydrophilic composition of interface and surface residues. The composition in terms of temperature factors (B-factors) is also considered as it was shown to have discriminative power between crystal contacts and biological interfaces^58^. All the different properties are weighted according to the coverage of the target sequence (i.e. the fraction of target residues mapped on the template). All features used in this study are explicitly defined in Supplementary Table S2.

Our dataset of models was divided in a train-test set (70%) and a validation set (30%). A 10-fold cross-validation in combination with a grid search was performed on the train-test set to fine-tune the SVM hyper-parameters and avoid overfitting. The resulting predictors were used to rank templates of the validation set. To assess the ability of the predicted QS-score to correctly rank the models we used an evaluation scheme in analogy to the one used in CAPRI^15^: the quality of models with a QS-score below 0.1 is deemed as “incorrect”, between 0.1 and 0.3 as “low”, between 0.3 and 0.7 as “medium”, and higher than 0.7 as “high”. For each validation target the model generated from the top scoring template, in terms of predicted QS-score, was compared to the reference structure and assigned to one of the quality categories.

**Figure 6:**
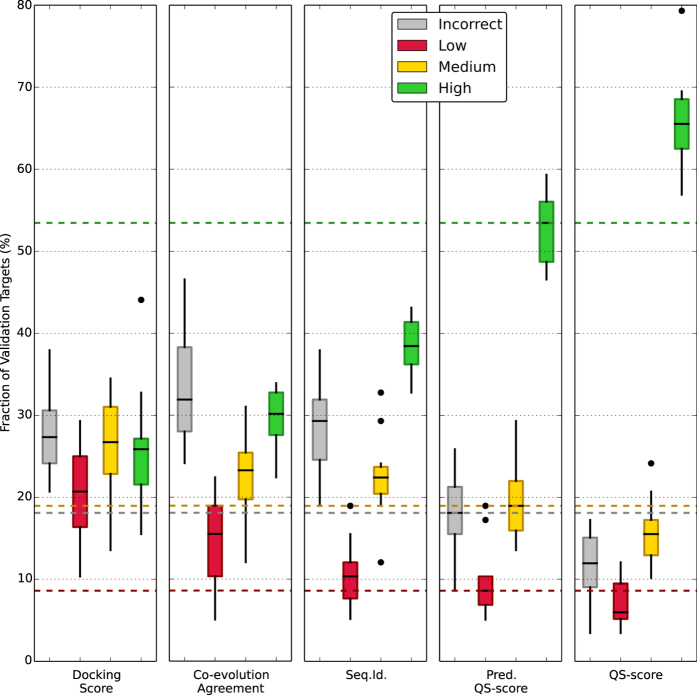
Fraction of top scoring models in each quality category using different ranking criteria. The evaluation scheme “incorrect” (QS-score < 0.1), “low” (0.1 ≤ QS-score < 0.3), “medium” (0.3 ≤ QS-score < 0.7) and “high” (QS-score > 0.7) resembles the scheme used in CAPRI measures. Five ranking criteria are considered: a physics-based docking score (Docking Score), the co-evolution predicted contact agreement (Co-evolution Agreement), the naïve sequence identity (Seq.Id.), our SVM prediction (Pred. QS-score) and the hypothetical “perfect” ranking based on the QS-score distance from the native structure (QS-score). The fraction of validation target is computed for the ten different cross-validation iterations.

The results are summarized in Fig. 6 where the SVM-predicted QS-score is compared to other ranking criteria: (i) a physics-based docking score as described in ref. 59, (ii) a co-evolution based score representing the agreement between models and GREMLIN^60^ predicted contacts (see “Co-evolution Agreement” in Material and Methods), (iii) a sequence identity criteria that would always rank first the model whose template has the highest sequence identity to the target sequence, (iv) the QS-score criteria, that ranks models according to their distance from the native structure (i.e. the perfect but hypothetical ranker). Looking at the latter criterion, we observe that a considerable fraction of the validation targets can be modeled with high quality (median of 65%). Ranking models by docking interaction energy proved unsuccessful, selecting high quality models sporadically (median of 25%). Using contact predictions based on co-evolution has been shown to be useful in *de novo* modelling^61^ and discriminating interacting and non-interacting partners in multimeric complexes^14^. Here, however, we show that it is not providing enough information to choose between alternative quaternary structures (high quality fraction median of 30%) within a family of proteins. The naïve idea of selecting the models with highest sequence identity provides high quality models in only 39% of the cases. Our SVM prediction approach improves the ranking significantly with a median of 52%. This improvement is highlighted by the lower fraction of incorrect models.

To characterize the relative importance of each feature we trained predictors using only single features (Supplementary Figure S2). Most of the descriptors based on sequence can correctly rank 45% of the validation targets, followed by PPI fingerprint features at 35%. Analyzing the correlation of the features (Supplementary Figure S3) it is clear that sequence derived features form a cluster which is not correlated to the PPI fingerprint features. This indicates that PPI fingerprint features are bringing novel information to the predictor. A minor optimization of the feature set is possible by selecting only the top performing features with univariate linear regression tests. Using the top 25 features gives the best performances in our cross-validation experiment (Supplementary Figure S4). Two out of three discarded features are about accessibility agreement (surface, and core regions) while the last one is the average profile entropy. The top five features selected are related to interface and its conservation: sequence identity, similarity and secondary structure agreement of the aligned interface fraction and the PPI fingerprint curve in terms of its area and absolute maximum. This confirms that PPI fingerprint analysis provides valuable information for quaternary conformations prediction.

**Figure 7:**
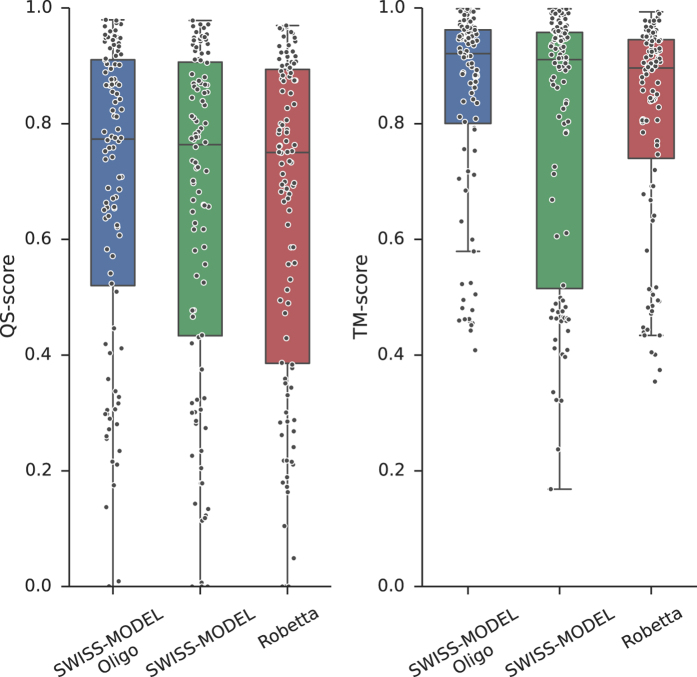
Comparison of model quality for three servers participating in CAMEO. The approach described in the current study (SWISS-MODEL Oligo) is compared to the classic SWISS-MODEL and Robetta servers. Common set of 111 homo-oligomeric models produced by all servers is compared to the native structure using two distance measures: QS-score (representing interface accuracy) and TM-score (representing global fold accuracy).

An additional validation set is provided by the Continuous Automated Model EvaluatiOn performed by CAMEO^32^. The CAMEO server retrieves on a weekly basis the sequences of new PDB entries that will be released the following week. The sequences are submitted to several structure prediction servers and, when the actual structure is published, the models are evaluated. Not many publicly available servers perform quaternary structure prediction. We could analyze the quality of models produced by the classical SWISS-MODEL server^52^ and Robetta^62^. A modified version of the SWISS-MODEL server including the pipeline presented in this study (SWISS-MODEL Oligo) was used for a retrospective analysis running the template search on corresponding previous releases of the PDB. We compared models produced by these servers from August 2015 to August 2016. The predictions of these three servers had a total of 111 common homo-oligomeric targets. The models produced by each server are compared to the native structure using QS-score and a structural-similarity based measure, TM-score, obtained using MM-align^46^ after the subunits were correctly mapped and chains renamed. The method we propose outperforms the other servers in terms of interface quality (QS-score) and in global structural similarity (TM-score) without being explicitly trained on this last distance measure (Fig. 7). Our approach is also able to better detect whether to model an oligomer or a monomer, showing no tendencies to over-predict oligomers (Supplementary Table S3).

### Application example: modeling of fructose bisphosphate aldolase complexes

Fructose bisphosphate aldolase (FBA) is an enzyme catalyzing a central step in the glycolysis pathway by splitting the hexose ring of fructose 1,6-bisphosphate (FBP) into two triose sugars: glyceraldehyde 3-phosphate (GAP) and dihydroxyacetone phosphate (DHAP). FBAs are divided into two classes depending on their mechanism of action: class I aldolases form reaction intermediates by covalently linking the DHAP to a conserved lysine in the active site; class II aldolases instead rely on the presence of a metal cofactor^63^. The quaternary structure of class I aldolases (found mostly in eukaryotes) is homo-tetrameric, while class II aldolases (found in prokaryotes and lower eukaryotes) can assemble in different stoichiometries the most common being homo-dimer or homo-tetramer^64–66^.

**Figure 8:**
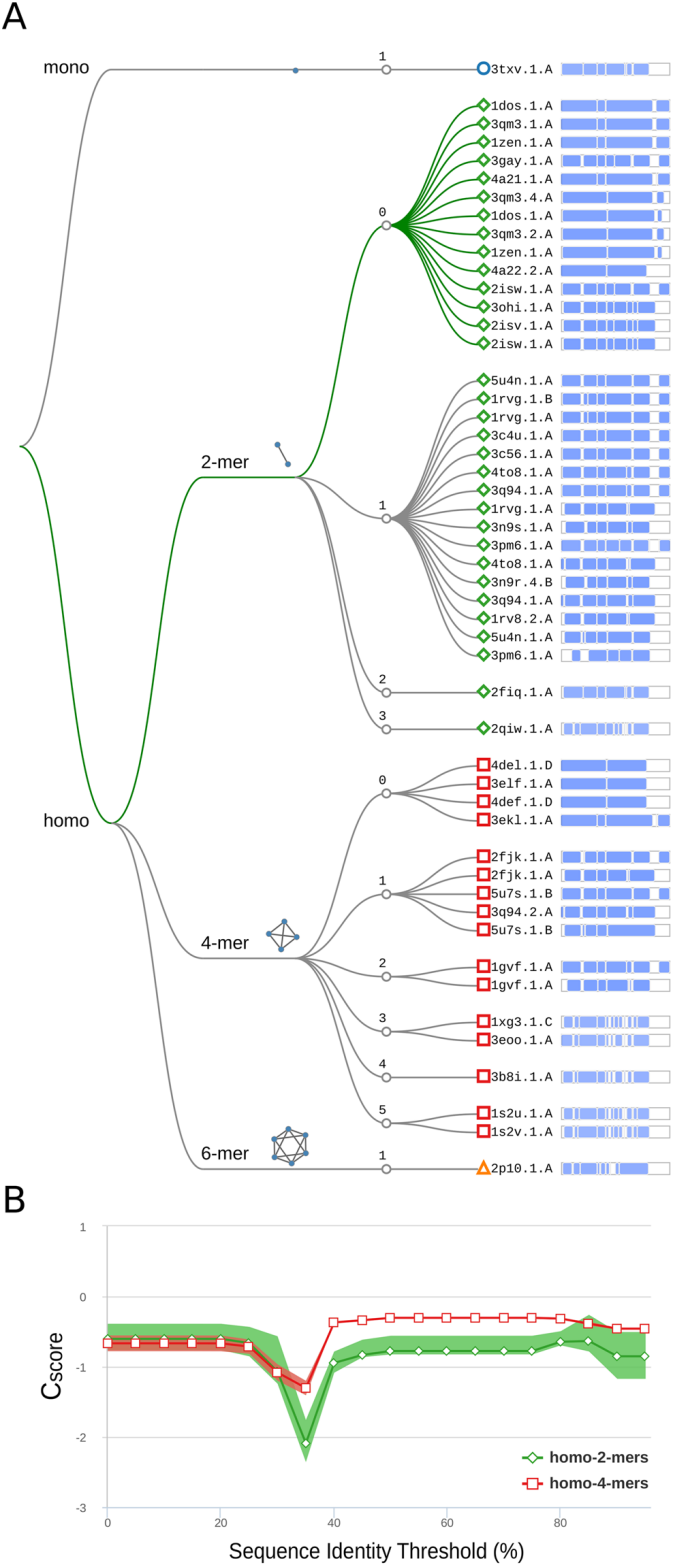
Quaternary structure analysis *of H.volcanii* fructose bisphosphate aldolase (FBA). (**A**) Structural clustering tree *of H.volcanii* FBA homologs with known structure. Each leaf is a template labeled with the PDB code and a bar indicating sequence identity and coverage (darker shades of blue refer to higher sequence identity). The decision tree follows the described levels of clustering: oligomeric state, stoichiometry (the topology of the complexes is also shown), and QS-score clustering. The green thread indicates templates with a predicted conserved QS. (**B**) The PPI fingerprint curves of the dimeric (green) and tetrameric (red) sets (the area plot spans between the 25^th^ and 75^th^ percentiles). The dimeric forms of FBA have a stronger interface conservation signal with respect to the tetrameric form. This stronger conservation is observable using different evolutionary distance thresholds, notably taking into account the entire MSA would not highlight a diverse conservation pattern.

We illustrate the application of our approach on the example of a class II FBA from *Haloferax volcanii* (UniProt AC: D4GYE0). No crystal structures of this specific enzyme or of homologs having closely related amino acid sequence are available. The result of structural template clustering is reported in Fig. 8A in a decision tree style. Sequence identity highlights two clusters of dimeric and tetrameric templates, but does not allow for a finer differentiation as all the highlighted templates span the range between 25–35%. A more indicative feature is the PPI fingerprint curve for these two groups (Fig. 8B). The dimeric and tetrameric interfaces follow two different patterns. The conservation score obtained using a complete MSA is almost equal for both the dimeric and tetrameric options, with tetramers being slightly more conserved. The minimum for both the curves is between 30% and 40% sequence identity which is the typical distance between most of the FBAs. From this minimum to higher sequence identity thresholds the indication for dimeric interface conservation is stronger reaching lower absolute values. Even in absence of direct structural evidence, we can thus state that the dimeric interface is more conserved than the tetrameric one among close homologs of the target protein. The SVM QS-score predictor is able to capture the discussed trend and assign a higher score to dimeric templates (predicted QS-score higher than 0.5 are indicated by the green thread on the decision tree). This protein was indeed proven to be homo-dimeric^67^ by gel filtration chromatography and molecular weight consideration. Notably, no aldolases were included in training or validation set; nonetheless our predictor is able to generalize on this unseen protein family and correctly assigns high predicted QS-scores to dimeric templates. This example illustrates how the quaternary structure of proteins can be inferred with high confidence.

## Conclusions

Developing a new protein interface distance measure which considers the entire complex interface allowed us to glance at the surprising heterogeneity of the multimeric protein structure space. Aloy *et al*.^33^ noted that binary domain-domain interactions are structurally conserved above 30–40% sequence identity and Levy *et al*.^34^ noted that the symmetry of the complexes is almost invariably conserved over 90% sequence identity. In agreement with these analyses we observe that the majority of close sequence neighbors retain the same quaternary structure and binding mode. Nonetheless, in one third of the cases where multiple different assemblies are observed for similar sequences, sequence similarity is not a safe proxy for similar quaternary structure. This highlights the necessity of explicitly considering all alternative quaternary structure conformations during the template identification step in homology-based modeling approaches.

Our findings on the behavior of interface conservation expressed as a function of evolutionary distance (PPI fingerprint) are in agreement with the results obtained by Duarte *et al*.^43^ where, for the purpose of discriminating crystal contacts and biological contacts, they identify a sequence identity threshold around 50–60%. Using the complete profile, however, provides a fine-grained description of protein family interaction landscape. This information, orthogonal to interaction energy considerations, helps in the differentiation between biologically relevant interactions and crystal contacts. When the PPI fingerprint concept is applied to homology modeling, it provides additional criteria to support one quaternary structure hypothesis over another, as illustrated in the FBA example.

Comparative modeling of the complete architecture of homo- and hetero-oligomers starting only from their amino acid sequences is feasible and effective. To our knowledge, this is the first attempt to predict protein assemblies for a large scale curated dataset taking into account their entire quaternary structure beyond binary interactions. The models produced with the described approach have a high-quality interface in 52% of the cases, which is halfway from the sequence identity baseline to the theoretical maximum given the current structural information in the PDB. The method we developed is publicly available at http://oligo.swissmodel.expasy.org and can aid molecular biologists and biochemists by providing an overview of homologs’ quaternary structural space along with the prediction made by our method. We are planning to extend the ranking approach presented here with single chain quality estimation in the next release of SWISS-MODEL.

The main limitation of our method is that of relying on available templates of homologous complexes. This is most evident in the case of hetero-oligomers where we could not identify templates for 20% of the initial dataset. Thanks to the large effort of structural biology, structures of macromolecular complex are continuously unveiled at unprecedented levels of detail. This will be reflected on our approach, enabling it to model more and more precise protein-protein interfaces and assemblies.

## Methods

### Conservation score

Conservation is expressed as Relative Entropy^35, 41, 68^:

1 $$R{E}_{c}={\sum }_{a}{p}_{a}{\mathrm{log}}_{2}\frac{{p}_{a}}{{p}_{ab}}$$

where *p*_*a*_ is the probability of an amino acid *a* to be in the alignment column *c* and *p*_*ab*_ is the background amino acid *a* probability distribution computed over the entire alignment (gaps are excluded).

The Relative Entropy (RE) is computed for each column *c* of a multiple sequence alignment and normalized in the interval [0, 1] with 0 indicating less conserved residues and 1 more conserved residues. The MSA is obtained running HHblits^42^ against the non-redundant (20% sequence identity) NCBI database with a threshold of 70% as minimum coverage. The MSA alignment is divided using 20 sequence identity inclusion thresholds (from 0% to 100% in steps of 5%). The column-wise RE is computed for each alignment.

We define the degree of conservation of an interface with respect to the surface using log-ratio of the average entropy of interface residues ${\langle S\rangle }_{i}$(weighted by relative solvent accessible surface area, *rASA*) over the average of those lying in the rest of the surface ${\langle S\rangle }_{s}$:

2 $$\langle S\rangle =\frac{\sum rAS{A}_{c}R{E}_{c}}{\sum rAS{A}_{c}}$$

3 $$IS=\,\mathrm{ln}\,\frac{{\rm{1}}+{\langle S\rangle }_{i}}{{\rm{1}}+{\langle S\rangle }_{s}}$$

A negative interface-surface ratio (*IS*) between interface entropy distribution and surface entropy distribution indicates that residues placed in the interface are less prone to mutate when compared to surface residues. Eventually, the interface-surface ratio is normalized by the number of interfaces involved.

To test the significance of the observed interface conservation we randomly sample “patches” of surface residues and compute their conservation (excluding the original interface residues). We define an adjacency graph of surface residues considering neighboring residues to have at least one atom within *N* Å apart each other (where *N* is dynamically set in order to obtain a connected graph). A surface residue is randomly picked and neighbors are added until the number of residues of the patch equals that of the interface. This process is iterated *n* times (where *n* is proportional to the original surface size). At each iteration, surface residues not included in the patch are used to evaluate the interface-surface ratio, resulting in a distribution *X* = (*x*_1_, …, *x*_*n*_) of ratios. We can estimate the P-value of the original interface as:

4 $$P=min({\int }_{{\rm{\min }}(X)}^{IS}{\hat{f}}_{h}(X)dX,{\int }_{IS}^{{\rm{\max }}(X)}{\hat{f}}_{h}(X)dX)$$

where *IS* is the native interface’s interface-surface ratio and ${\hat{f}}_{h}$ is a kernel density estimated probability density function with a bandwidth parameter *h* computed using Silverman’s rule of thumb.

Finally the conservation score is:

5 $${C}_{score}=IS(1-P)$$

where the original interface-surface ratio *IS* is weighted by the P-value complement. So when an interface is close to the random patch distribution the score will tend to 0. The curve is numerically described by four features: i) the minimum (lowest value), ii) the absolute maximum (the highest value independently if negative or positive), iii) the value of the curve considering the full MSA, and iv) the area of the curve (computed as integral using the composite trapezoidal rule).

### QS-score Algorithm

The number of possible mappings between two complexes A and B having a different number of subunits is $$(\begin{array}{c}{n}_{A}\\ {n}_{B}\end{array})$$ where *n*_*A*_ is the number of chains in the larger complex A and *n*_*B*_ those of the smaller complex B. In the worst case of two equally sized complexes the number of possible mappings is *n*!. This becomes untreatable when comparing big complexes such as viral capsids. However, when symmetry information is available in the PDB coordinate information or can be deduced from the complex geometry, the problem can be reduced to the identification of the mapping between symmetry related groups, which are typically containing a number of treatable subunits. To our knowledge, this currently is the only algorithm taking into account the problem of chain mapping. The steps performed by the QS-score algorithm are the following:

1. Polypeptide chains within each complex are grouped by their chemical equivalence (e.g. the two α chains in human hemoglobin)
2. Equivalent entities between the two assemblies to be compared, are identified by global sequence alignment (e.g. hemoglobin chains α in two different structures)
3. Symmetry or pseudo-symmetry of each complex is calculated and chains which can be roto-translated reproducing the full assembly are grouped in symmetry groups (e.g. in hemoglobin two pairs of α-β chains)
4. The chain mapping between two symmetry groups in different assemblies is identified by superposition. This symmetry group mapping is applied to all symmetry groups.
5. For each symmetry group of step 3 all possible pairs are considered
  - A symmetry group pair is used as base to superpose complexes
  - The lowest global RMSD highlight the correct mapping
6. Equivalent residues between the assemblies are indexed by sequence alignment.

From the inter-complex chain mapping we can deduce also the inter-complex residue mapping by aligning the sequences of each chain in the complexes. Each residue in the first complex that can be mapped to a residue in the second complex (and vice-versa) is included the set of “mapped” residues. We consider an interface contact to occur when Cβ atoms (Cα for Glycine) of pair of residues belonging to different chains are at most 12 Å apart. This definition of contact is inspired by Q-score and it allows us to compare structures not having identical side chains. Pairs of contacts (one for oligomer A and one for oligomer B) are defined as “shared” when all residues involved are “mapped”. Residue pairs that form contacts but are not “mapped” or that are “mapped” but form contacts only in one of the oligomers, are defined as “non-shared”.

QS-score is then defined as follow:

6 $$\mathrm{QS}-\mathrm{score}=\,\frac{{\sum }_{{\rm{shared}}({\rm{A}},{\rm{B}})}{\rm{w}}({\rm{\min }}({{\rm{d}}}_{{\rm{A}}},{{\rm{d}}}_{{\rm{B}}}))\,(1-\frac{|{{\rm{d}}}_{{\rm{A}}}-{{\rm{d}}}_{{\rm{B}}}|}{12})}{{\sum }_{{\rm{shared}}({\rm{A}},{\rm{B}})}{\rm{w}}({\rm{\min }}({{\rm{d}}}_{{\rm{A}}},{{\rm{d}}}_{{\rm{B}}}))+{\sum }_{non-shared(A)}w({d}_{A})+{\sum }_{non-shared(B)}w({d}_{B})}$$

where *d* is the Euclidean Cβ distance between the residues, the second term at the numerator is the relative error (considering 12 Å as maximal error) and *w* the weighting function:

7 $$w(d)=\{\begin{array}{lll}1 & if & d\le 5\\ {e}^{-2{(\frac{d-5}{4.28})}^{2}} & if & d > 5\end{array}$$

which expresses the probability of a side-chain interaction given the Cβ distance as derived by Xu *et al*.^44^ fitting a half-gaussian model to observed sidechain contacts. If oligomer A and oligomer B have only “shared” contacts and all the distances are identical, QS-score is 1, indicating identical interfaces. When the distances are not equal, the relative error factor will push the QS-score towards 0 proportionally to the difference in the distances. The same happens in case of “non-shared” contacts.

### Interface definition

We compute the accessible surface area (ASA) of the monomer and the buried surface area (BSA) of the assembly with the Naccess implementation of the Lee-Richards algorithm^69^. Following the definitions of interface core and surface residues in ref. 70, we define surface residues as those having a relative accessibility (rASA) larger than 25% considering the monomer; interface residues are those whose relative buried surface area (rBSA) is higher than 25% and that have a rASA below 25% when considering the assembly; the remaining residues are considered as protein’s core residues.

### PDB-wide QS clustering

All homo- and hetero-oligomeric structures deposited in the PDB where considered. Chains consisting of small peptides (below 20 amino acids) or Cα traces were excluded, and in case only a single chain remained after filtering, this was also ignored. This resulted in 90,764 assemblies for 63,902 PDB entries and 356,585 polypeptide chains. The chain sequences where clustered using CD-HIT^71^ (90% sequence identity). To properly handle heteromeric structures (different chains of a PDB entry may appear in different clusters), a sequence cluster is defined as the set of chain clusters IDs to which each chains of the complex is belonging. This resulted in 24,272 clusters of which 13,896 (57%) included multiple assemblies and were hence further analyzed. The assemblies in each sequence cluster were compared using QS-score and the resulting distance matrix was used to perform a hierarchical/agglomerative clustering using complete linkage. 491 clusters (3% of the total number of clusters) were excluded mostly due to incompatible symmetry groups between the compared assemblies which led to an intractable number of possible mappings.

### TARGET Dataset

The homo-oligomer dataset is derived from the PiQSi database^72^. PiQSi comprises ~20,000 annotated biological units which we reduced culling the sequences with PISCES^73^ on a 25% sequence identity basis. We visually inspected entries with multiple binding modes to select those which are described in the respective paper. For hetero-oligomers we started from the complete list of PDB entries annotated as hetero-complexes. As an initial filter we removed complexes which are marked as hetero-oligomers because of their interaction with antibodies or short peptides (below 20 amino acids). We filtered out complexes with an average per interaction BSA below 250 Å^2^ and having unconnected components. We then culled the set in order to get high quality representatives of unique interactions (with a resolution of at least 3.0 A). To reduce the redundancy we clustered the subunits’ sequences by a 30% sequence identity threshold using CD-HIT^71^ and we grouped complexes whose chains belonged to the same set of clusters. We kept only the most inclusive assemblies (i.e. sub-complexes were discarded). Finally, we structurally clustered the complexes using CATH^74^ domains annotation retaining only those which had a unique set of domains at the topological level.

### MODEL Dataset

This dataset consists of homology models based on the alignment of the target sequence to template structures generated with PROMOD3 (Studer *et al*., in preparation), a comparative modelling engine based on OpenStructure^75^. The loop candidates are selected with a database approach and are then adapted to the environment using CCD^76^ and a final candidate gets selected using statistical potentials of mean force. The sidechain modelling is inspired by SCWRL4^77^. A final energy minimization is performed using the OpenMM molecular mechanics library^78^. Each model is annotated with the QS-score to the native structure and the set of features described in the text. To ensure an un-biased learning step, all models are grouped by target. This way, during cross-validation, the set of targets can be randomly divided in testing and validation sets avoiding similar models of a same target to be used at the same time for testing and validation.

### Clustering homologous assemblies

Several databases^45, 47, 79–82^ target the problem of grouping similar interactions. For example, in the ProtCID^47^ database interfaces are grouped depending on PFAM domains architectures. While ProtCID is a great tool to compare interface of homologous proteins found in different crystal forms, it accounts only for binary interactions. The first database which specifically addresses entire assemblies is 3D Complex^79^. The classification implemented in 3D Complex is based on the reduced representation of biological assemblies as graphs and it relies on SCOP domain architecture to define similar interactions. Our aim is to cluster homologous assemblies, which are expected to be redundant in terms of domain architecture, but which can be diverse from an atomistic point of view. Hence, we defined a hierarchical clustering scheme aware of entire complex topology as well as interatomic contacts occurring at the interface. The clustering is based on hierarchical levels which represent structural organization of biological complexes. The fraction of templates in each cluster (compared to the total number of identified templates) is measured in the consensus features.

The first level describes the nature of the interacting subunits and is characterized by three possible states: we distinguish templates composed by a single polypeptide chain, labeled as “mono”; templates composed by two or more different chains, labeled as “hetero”; templates with two or more identical chains, labeled as “homo”. The second level is based on the stoichiometry of the complex, so the amount of chains with a specific sequence. Finally, the last level clusters templates using a greedy hierarchical clustering approach based on QS-score distance measure.

### Co-evolution agreement

GREMLIN^60^ was used to predict contacts by co-evolution analysis. We computed a co-evolution score in the form of an agreement score between the predicted inter-chain contacts and the models we generated. The co-evolution score is computed as the number of predicted contact found in a model (Cβ-Cβ distance < 7 Å, Cα for glycine) over the total number of predicted contacts (maximum 1.5 times the length of the target sequence/s). Hence, a co-evolution agreement close to 1 indicates a perfect agreement while a value close to 0 indicates that no predicted contacts are found in the model. Following the GREMLIN protocol, we were not able to obtain alignments of sufficient depth for every protein sequence in our dataset. Out of a total of 818 unique possible binary interactions (362 homomeric, 456 heteromeric) in our dataset, we obtained a contact prediction in 549 cases (290 homomeric, 259 heteromeric). While for homomeric targets an inter-chain contact prediction is very likely to succeed (99% of the cases), inter-chain contacts prediction were not always available for heteromers (34% of the cases). For heteromeric multimers all the pairwise combinations of paired alignments were performed as done by Ovchinnikov *et al*.^83^.

### Data availability statement

Data sets generated and analysed during this study are included in this published article and its Supplementary Information files. Intermediate data (alignments, models) of current study are available from the corresponding author on reasonable request^15, 48–51^.

## Electronic supplementary material

Supplementary Information

Dataset
