# Supplementary material for: Modeling protein quaternary structure of homo- and hetero-oligomers beyond binary interactions by homology
Source: Sci Rep. 2017 Sep 5;7:10480. doi: 10.1038/s41598-017-09654-8 (PMC5585393; doi:10.1038/s41598-017-09654-8)
Supplement: Supplementary file 1 — Supplementary Information [file 41598_2017_9654_MOESM1_ESM.pdf]

Title

Modeling protein quaternary structure of homo- and hetero-oligomers beyond binary interactions by homology

Author Affiliation

Martino Bertoni [1,2], Florian Kiefer [1,2], Marco Biasini [1,2], Lorenza Bordoli [1,2], Torsten Schwede [1,2]

[1] SIB Swiss Institute of Bioinformatics, Basel, Switzerland

[2] Biozentrum, University of Basel, Klingelbergstrasse 50/70, 4056 Basel, Switzerland

Supplementary Table S1. Interface distance measures developed in the last years. For each we report the measure name, the reference paper, whether is suitable for binary interfaces or multimeric interfaces and a short summary of the method.

**Measure**	**Reference**	**Binary**	**Multimeric**	**Method Summary**
f_{nat}	CAPRI assessment [43-47]	X	-	Fraction of correctly predicted contacts
L_rms		X	-	RMSD of ligands (smallest chains)
I_rms		X	-	RMSD of interface atoms
iRMSD	Aloy *et al* [33]	X	-	RMSD calculated on 14 predefined coordinates (independent chain superposition)
iTM-score	Gao and Skolnick [46]	X	-	Geometric distance of interface residues
IS-score	Gao and Skolnick [46]	X	-	Contacts similarity of interface residues
MM-align	Mukherjee and Zhang [48]	X	X	Structural alignment by chain-joining
Q-score	Xu et al [49-51]	X	-	Geometric distance differences between equivalent interfacial residue

Supplementary Table S2. Summary of the features used in this study. For each feature the group to which is belonging, its name and its definition are provided.

Feature Group	Feature Name	Definition
Sequence	Sequence Identity	The fraction of identical residues divided by the total number of aligned residues in the target-template alignment (gaps are ignored).
	Sequence Similarity	Given two aligned sequences A, B: $$sim(A,B) = \frac{1}{L}\sum_{i=1}^{l} M(a_i, b_i) \qquad (S1)$$ Where L is the number of columns in the alignment (gaps are ignored). $$M(a,b) = \begin{cases} \frac{m(a,b) - \min(m)}{\max(m) - \min(m)} & \text{if a} \neq \text{gap and b} \neq \text{gap} \\ 0 & \text{otherwise} \end{cases} \qquad (S2)$$ Where *m(a,b)* are the BLOSUM62 scores, *min(m)* and *max(m)* are the lowest and highest scores available in the substitution matrix.
	Secondary Structure Agreement	Predicted secondary structure is computed for the target and the template (with PSIPRED), and the one letter code states are mapped on the target-template alignment. The agreement is computed as the fraction of matching secondary structure states, over the total number of aligned residues.
	Accessibility Agreement	Analogous to “Secondary Structure Agreement”, but predicted solvent accessibility (with SSpro4) is used.
	Surface Sequence Identity	Analogous to “Sequence Identity”, “Sequence Similarity”, “Secondary Structure Agreement”, and “Accessibility Agreement” respectively. Only residues belonging to the surface (see “Interface Definition” in Material and Methods) of the template are considered.
	Surface Sequence Similarity	
	Surface Secondary Structure Agreement	
	Surface Accessibility Agreement	
	Core Sequence Identity	Analogous to “Sequence Identity”, “Sequence Similarity”, “Secondary Structure Agreement”, and “Accessibility Agreement” respectively. Only residues belonging to the core (see “Interface Definition” in Material and Methods) of the template are considered.
	Core Sequence Similarity	
	Core	

	Secondary Structure Agreement	
	Core Accessibility Agreement	
	Interface Sequence Identity	Analogous to "Sequence Identity", "Sequence Similarity", "Secondary Structure Agreement", and "Accessibility Agreement" respectively. Only residues belonging to the interface (see "Interface Definition" in Material and Methods) of the template are considered.
	Interface Sequence Similarity	
	Interface Secondary Structure Agreement	
	Interface Accessibility Agreement	
Multiple Sequence Alignment	PPI Fingerprint minimum	The interface and surface residues of a template are mapped on the target's MSA. The lowest value in the PPI Fingerprint curve (calculated as in "Conservation Score" in Materials and Methods) is considered.
	PPI Fingerprint absolute maximum	Analogous to "PPI Fingerprint minimum", but the highest value of the absolute (modulus) PPI Fingerprint curve is considered.
	PPI Fingerprint full MSA	Analogous to "PPI Fingerprint minimum", but the value of PPI Fingerprint curve at 0% sequence identity inclusion threshold (the complete MSA) is considered.
	PPI Fingerprint Area	Analogous to "PPI Fingerprint minimum", but the area of the PPI Fingerprint curve (integral of the curve using the composite trapezoidal rule) is considered.
	Profile Average Entropy	Arithmetic mean of column entropies in the HHblits generated MSA. Column entropy is defined as: $$H = -\sum_{a} p_a \log(p_a) \qquad (S3)$$ On all amino acids a in the column and p_a is the frequency occurrence of that amino acid in the column.
	Profile E-value	The $\log_{10}$ E-value returned by HHblits.
QS consensus	Oligomeric State Consensus	Given a template and the set of templates identified during the search step, the oligomeric state consensus is the fraction of templates, in the template search, sharing the same oligomeric state with the templates of interest.
	Stoichiometry Consensus	Analogous to the "Oligomeric State Consensus", but expressing the fraction of templates having the same stoichiometry as a template of interest.
	Interface	Analogous to the "Oligomeric State Consensus", but expressing

	Similarity Consensus	the fraction of templates having a structurally similar interface as a template of interest. Templates with similar interfaces are defined as those having a QS-score > 0.8.
Composition	Hydrophilic Propensity	Interface propensity give hydrophilic residues (D, N, E, Q, R) composition in the template interface vs. the surface expressed as: $$P_{phi} = ln\frac{1 + p_{phi,I}}{1 + p_{phi,S}} \qquad (S4)$$ Where $p_{phi,I}$ are $p_{phi,S}$ are the fraction of hydrophilic residues in the interface and surface respectively.
	Hydrophobic Propensity	Analogous to "Hydrophilic Propensities", but considering hydrophobic residues (I, L, V, F, M, A, G).
	Average B-factor Ratio	The log odd ratio between the average B–factor of interface and surface residues. $$B_{IS} = ln\frac{1 + \langle B \rangle_I}{1 + \langle B \rangle_S} \qquad (S5)$$

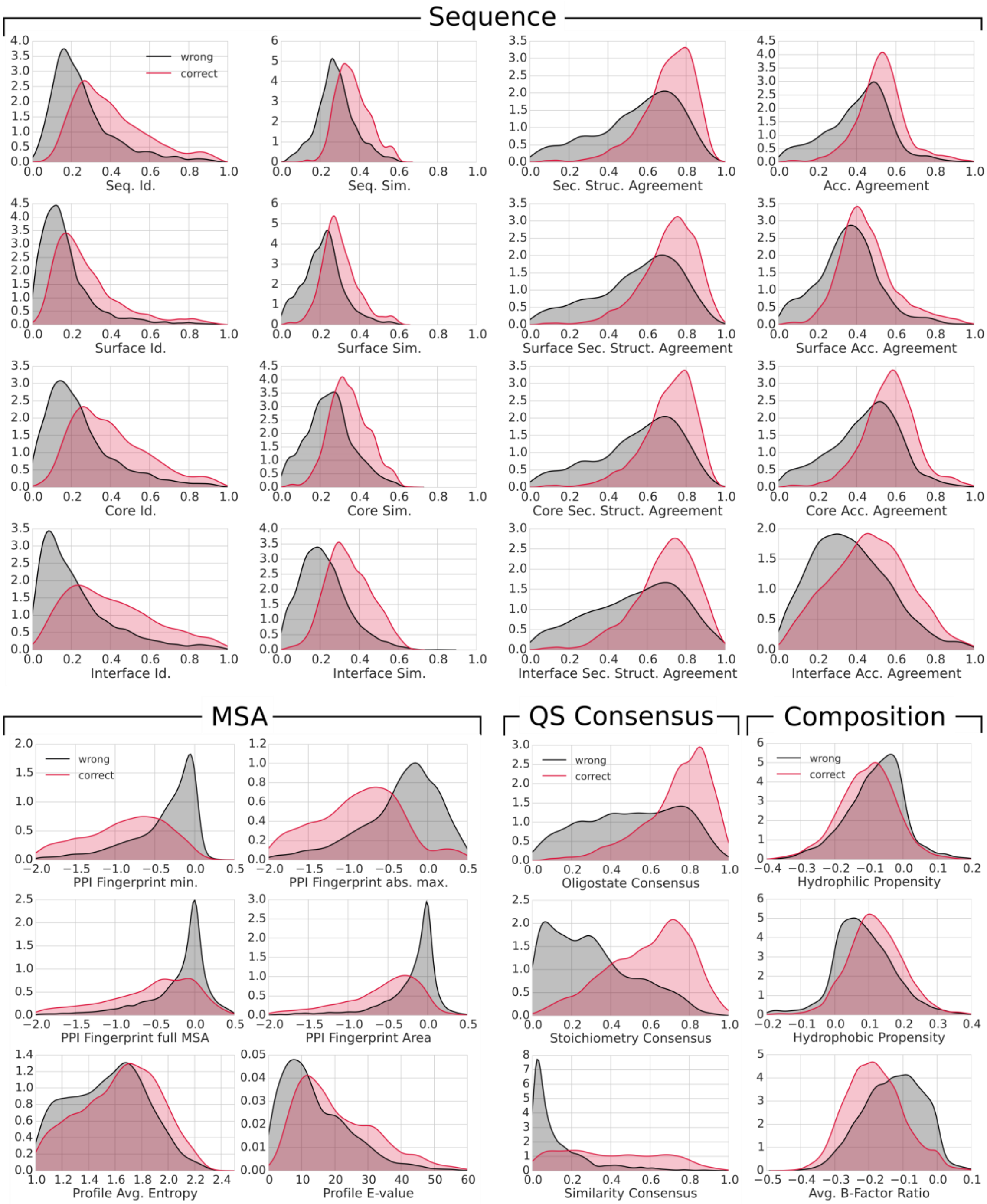

Supplementary Figure S1**.** Distribution of mostly correct (red) and mostly incorrect (black) models for different template features used in this study. Mostly correct models are those having a QS-score with the known target structure of ≥ 0.5 and mostly incorrect models are those with QS-score < 0.5.

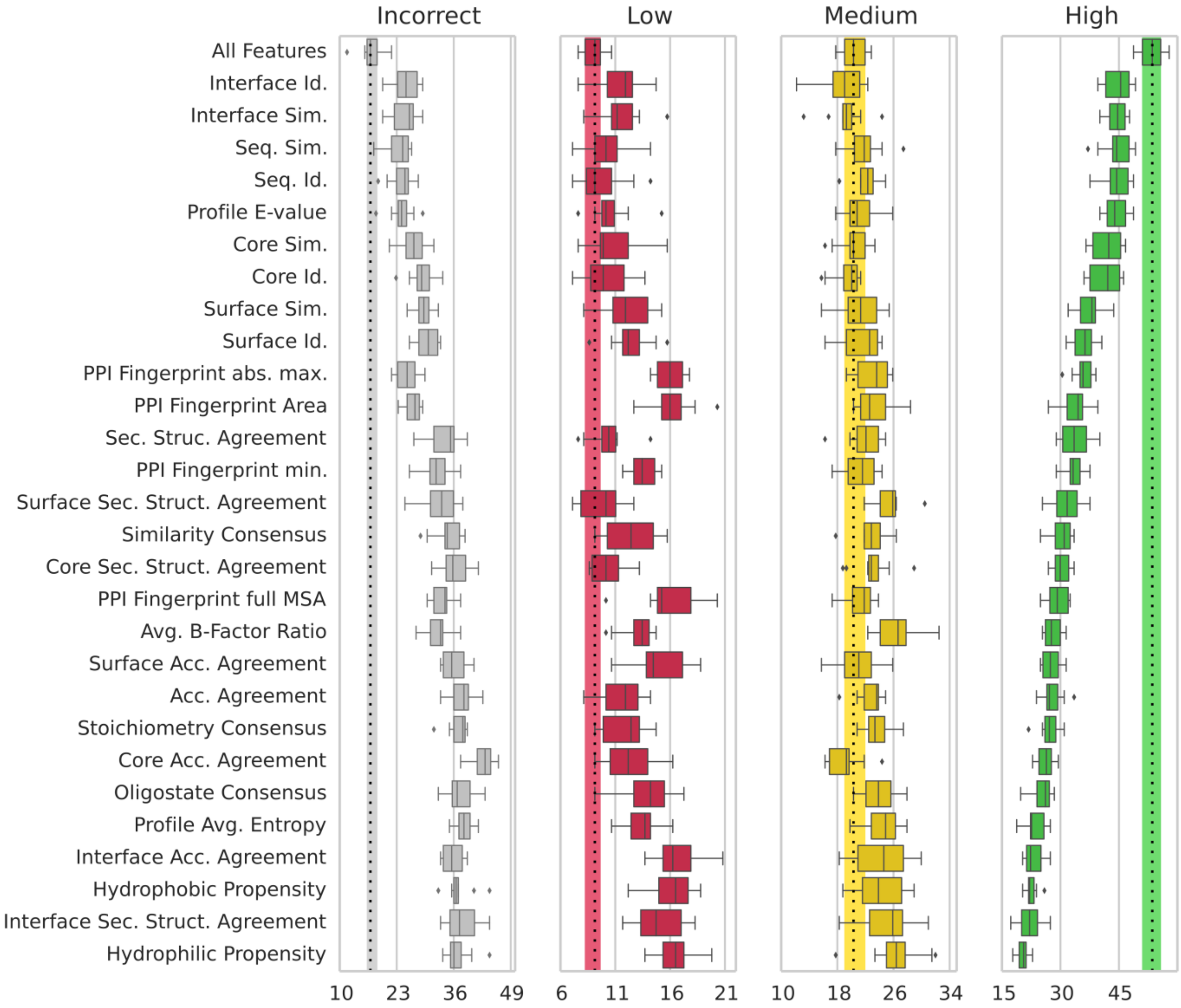

Supplementary Figure S2. Fraction of top scoring models (x-axis) in each quality category using single features. As reference the result obtained using all the features is reported and the vertical bar spans from the 25th to the 75th quartile with the median highlighted by a vertical dashed line. The evaluation scheme is based on the comparison of the top ranked model in comparison to the native structure: “incorrect” (QS-score < 0.1), “low” (0.1 ≤ QS-score < 0.3), “medium” (0.3 ≤ QS-score < 0.7) and “high” (QS-score > 0.7). The features are sorted in descending order based on the median of the high quality category performance.

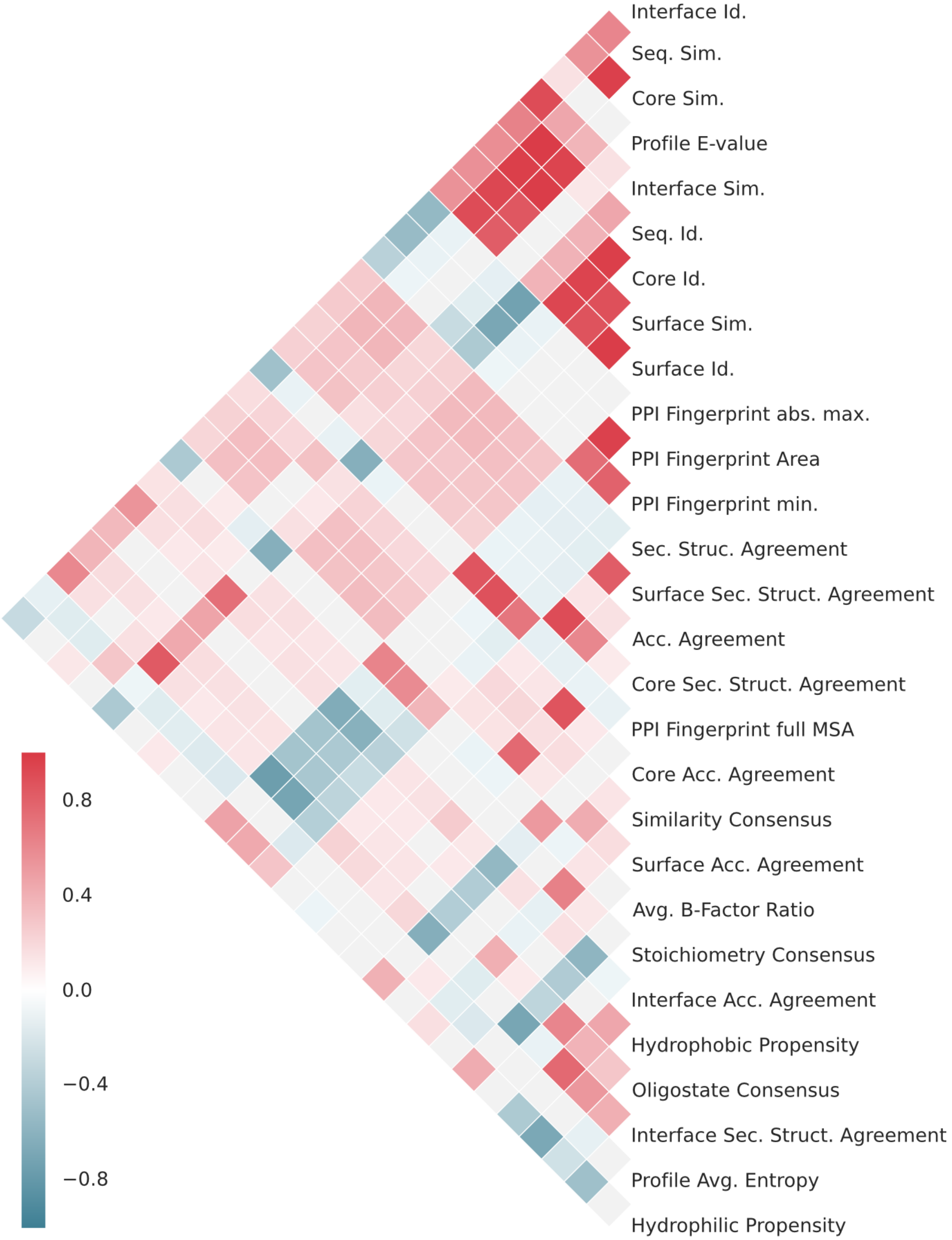

Supplementary Figure S3. Feature correlation plot. Each square of the triangular matrix represent the spearman correlation between pairs of features.

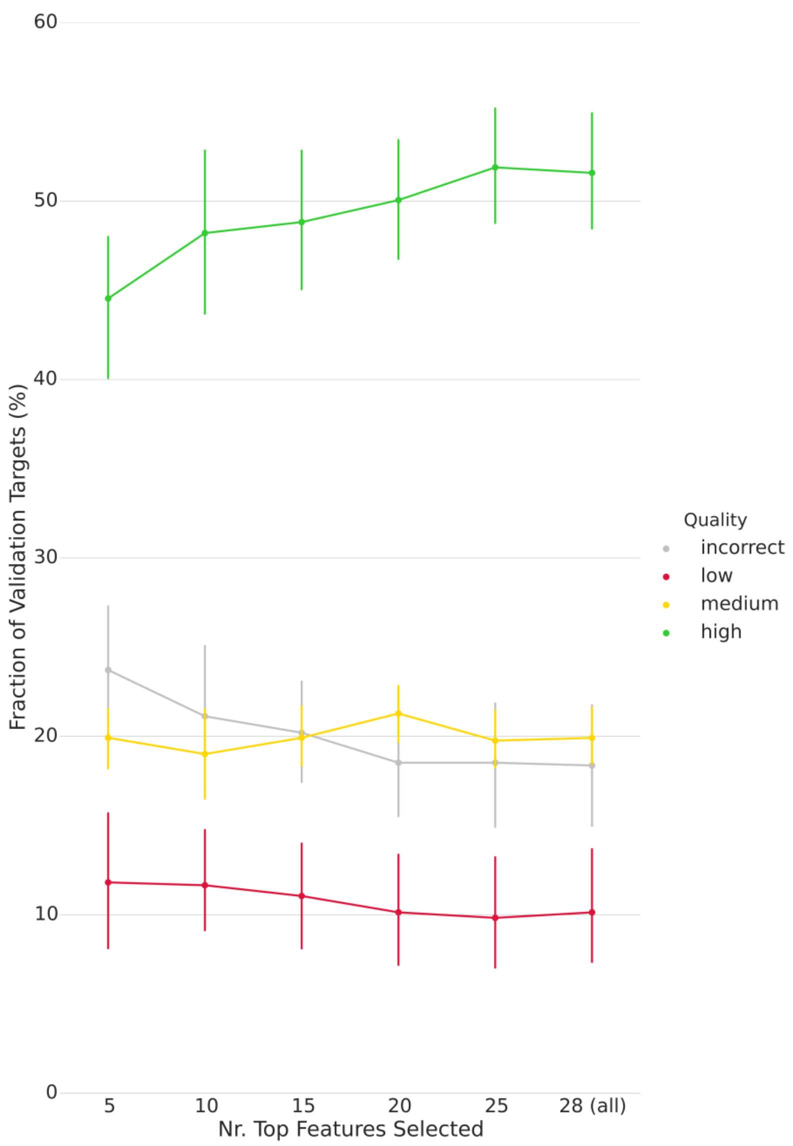

Supplementary Figure S4. Univariate feature selection. We analyzed the performances of different predictors trained with a subset of the original feature compared to the full set ("All Features"). Top 5, 10, 15, 25 features are selected by univariate linear regression tests. For each regressor we show the fraction of selected models falling in the different quality criteria described in the main text. The 95% confidence intervals are reported. Including more features result in a higher fraction of high quality models and lower fraction of incorrect, low and medium quality models.

Supplementary Table S3. Summary of the modeling performances of SWISS-MODEL Oligo (the server based on the current study), SWISS-MODEL, and Robetta. From 2015-07-31 to 2016-08-01 a total of 813 targets (427 monomeric and 386 homomeric) have been submitted by CAMEO to these servers. For each server we report the number of models returned, the number of true positives (i.e. the target is homomeric and the model as well), false positives (i.e. the target is monomeric but is predicted as oligomeric), true negatives (i.e. the target is a monomer and also the prediction is a monomer), false negatives (i.e. the target is an oligomer but the prediction was monomeric). The percentages refer to the targets modeled by each server. In the last column the Matthews correlation coefficient is reported.

	**Models**	**TP**	**FP**	**TN**	**FN**	**MCC**
**SWISS-MODEL Oligo**	797	280 (35%)	92 (11%)	328 (41%)	97 (12%)	0.52
**SWISS-MODEL**	800	173 (21%)	28 (3%)	390 (48%)	209 (26%)	0.44
**Robetta**	789	167 (21%)	40 (5%)	379 (48%)	203 (25%)	0.40
